# Inhibitory Effect of Two Traditional Chinese Medicine Monomers, Berberine and Matrine, on the Quorum Sensing System of Antimicrobial-Resistant *Escherichia coli*

**DOI:** 10.3389/fmicb.2019.02584

**Published:** 2019-11-13

**Authors:** Tong Sun, Xiao-Dong Li, Juan Hong, Can Liu, Xin-Luo Zhang, Jin-Ping Zheng, Yuan-Jun Xu, Zheng-Yang Ou, Jing-Ling Zheng, Dao-Jin Yu

**Affiliations:** Fujian Key Laboratory of Traditional Chinese Veterinary Medicine and Animal Health, Fujian Agriculture and Forestry University, Fuzhou, China

**Keywords:** traditional Chinese medicine monomer, *Escherichia coli*, drug resistance, biofilm, quorum sensing

## Abstract

The quorum sensing (QS) system controls bacterial biofilm formation, which is highly related to the virulence and resistance of pathogens. In the present study, the effect of two traditional Chinese medicine (TCM) monomers, berberine and matrine, on biofilm formation and QS-related gene expression of antimicrobial-resistant (AMR) *Escherichia coli* strains was investigated by laser scanning confocal microscopy (LSCM) observation and real-time PCR. The results indicated a roughly positive relationship between biofilm formation ability and antimicrobial resistance. LSCM observation showed that berberine and matrine inhibited biofilm formation of AMR *E. coli* strains at 1/2 minimal inhibitory concentration (MIC) (1/2 MIC berberine at OD_630_: 0.1020; 1/2 MIC matrine: OD_630_: 0.1045); furthermore, abnormal cell morphology such as rounded and elongated cells was also observed. This finding was consistent with the downregulation of QS-related genes: *luxS*, *pfS*, *sdiA*, *hflX*, *motA*, and *fliA*. At 1/2 MIC and 1/4 MIC concentrations of berberine, a significant downregulation of *luxS*, *pfS*, *hflX*, *ftsQ*, and *ftsE* was observed. The results indicate that berberine and matrine can inhibit biofilm formation by inhibiting the QS system and that berberine is more effective than matrine.

## Introduction

The phenomenon of information transmission between chemical groups through chemical signals is called quorum sensing (QS) (Bassler et al., 1993). This process often occurs later in the stationary phase of bacteria (Rajput et al., 2016). The QS system plays an important role in bacterial resistance. Previous studies have shown that signaling molecules formed in *Pseudomonas aeruginosa* in the logarithmic growth phase increase the number of persister cells after treatment with ciprofloxacin (Moker et al., 2010). The formation of biofilms is related to the perception of bacteria about their surrounding environment and bacterial density (Kai, 2018). Bacterial biofilms lacking the QS system contain loosely packed cells with poor adhesion, resulting in an incomplete biofilm (Kumar et al., 2016). Therefore, some researchers speculate that biofilm formation in the QS system could be a potential drug target (Williams, 2002).

The formation of biofilms further facilitates the development and transmission of bacterial resistance (Fernandes, 2006). Compared with typical hereditary resistance, biofilm bacterial resistance shows reversibility and transitivity (Bernier et al., 2013); this is because the bacteria inside the biofilm are in a special living environment, wherein the horizontal transmission of drug resistance genes can occur between individuals, leading to easy spread of drug resistance (Lupien et al., 2015). Antibiotics are effective agents for treating bacterial infections. Because of the action of antibiotics on bacterial growth processes such as nucleic acid replication and cell wall biosynthesis, bacterial cells face tremendous pressure for survival, thereby resulting in the development of resistance (Sengupta et al., 2013). Thus, a substance that could attenuate the virulence and pathogenicity of bacteria without killing them, so that the immune system of the host could more effectively remove the bacteria without inducing selective pressure-driven resistance (Lupien et al., 2015), would be a breakthrough in the fight against bacterial resistance.

The QS system is not necessary for growth, and therefore, interference with the QS system will not affect the growth of individual bacteria; thus, the QS system could be a key target for solving the problem of virulence and drug resistance (Tay and Yew, 2013). With regard to interference with the QS system, some chemical synthesis inhibitors, including furanones, pyrrolidones, diketopiperazines, and AIP compounds, have already been investigated (Kline et al., 1999). However, the halogens in these inhibitors are somewhat toxic to humans. Because many such inhibitors are not used in chemotherapeutic drugs, the use of natural extracts as a QS inhibitor (QSI) is a better choice (Lee et al., 2011). At present, various traditional Chinese medicine (TCM) crude extracts and TCM monomers have been found to act as QSIs. Zhao et al. (2013) found that Yunnan Baiyao at the sub-inhibitory concentration of 2.5 mg/mL could downregulate the transcriptional levels of the *P. aeruginosa* genes *lasR*, *lasI*, *rhlI*, and *rhlR*, thus affecting biofilm formation. The flavonoids kaempferol and quercetin can inhibit the formation of *E. coli* O157:H7 biofilm (Vikram et al., 2010). Some researchers have found that medicinal plants such as garlic and dietary compounds such as areca and ginseng roots exhibit QSI activity (Bosgelmez-Tinaz et al., 2007). However, these studies mainly focused on the screening of TCM monomers as QSIs and paid less attention to the specific mechanisms of inhibition. Moreover, there are few studies on the inhibition of the *E. coli* QS system. Our previous studies revealed that QS is closely related to drug resistance and the release of virulence factors of *E. coli* in the aqueous environment; moreover, the formation of biofilm is also tightly linked with the regulation of the QS system (Lai, 2011). Therefore, it is very important to investigate the effects of TCM on the *E. coli* QS.

Berberine is an isoquinoline alkaloid and is a commonly used broad-spectrum antibacterial drug. It shows antimicrobial activity against viruses, fungi, protozoa, helminths, and various bacteria, including multiple pathogens and multidrug-resistant strains (Wang et al., 2015). Matrine is widely found in leguminous plants, bitter bean, broad bean root, and *Sophora flavescens* (Park et al., 2012), and it has been proven to have anti-inflammatory, antibacterial, antioxidant, immunomodulatory, and anticancer effects. Matrine is often combined with antibiotics to treat bacterial infections in animals. Previous studies have shown that it can reverse the resistance of *E. coli* to antibiotics, and its low toxicity characteristics are also considered to be safe for clinical treatment (Thompson et al., 2015). However, few studies have reported the effect of these two monomers on *E. coli* resistance and its QS. In the present study, berberine and matrine were selected to observe their inhibition of biofilm formation by drug-resistant *E. coli* and the expression of genes related to the QS system, and to investigate their mechanism of action. The study hopes to resolve the issue of *E. coli* resistance and provide a theoretical basis for future studies.

## Materials and Methods

### *E. coli* Culture

An antibiotic-resistant strain of *E. coli* was previously isolated and tested for its antibiotic resistance spectrum by Chen et al. (2018). The standard strain of *E. coli* ATCC 25922 was purchased from the National Institute of Pharmaceutical and Biological Products Control (Beijing, China). The cryopreserved *E. coli* standard strain ATCC 25922 and the isolated strain were regenerated by inoculation into a test tube containing 3 mL of Mueller-Hinton Broth and cultured to a logarithmic phase at 37°C in a constant temperature shaker. The concentration of the bacterial solution was adjusted to 0.5 M turbidity (a bacterial suspension with an OD_600_ between 0.08 and 0.10), and the solution was used as a seed liquid. After the strain was regenerated, the colonies were picked and inoculated into 3 mL of lysogeny broth (LB) medium, and the concentration of the bacterial solution was adjusted to approximately 0.5 M turbidity after shaking at 37°C, 190 r/min for 4 h. The TCM monomers were then added to the LB at the minimum inhibitory concentration (MIC) of 1 MIC, 1/2 MIC, 1/4 MIC, and 1/8 MIC, and a blank medium without the drugs was used as a control. Aliquots of the bacterial culture were collected every 1 h for a period of 24 h to measure the OD_600_ value.

### Antibiotic Resistance and TCM Monomer Sensitivity

Stock solutions of standard drugs were prepared at a concentration of 512 μg/mL. These drugs included ampicillin (CAS: 7177-48-2), cefazolin (CAS: 25953-19-9), cefotaxime (CAS: 64485-93-4), gentamicin (CAS: 1403-66-3), ciprofloxacin (CAS: 85721-33-1), tetracycline (CAS: 60-54-8), and chloramphenicol (CAS: 56-75-7).

The berberine standards (CAS: 2086-83-1) and matrine standards (CAS: 519-02-8) (purchased from the National Institute of Pharmaceutical and Biological Products Control, Beijing, China) were dissolved in autoclaved double distilled water to prepare a drug solution with a concentration of 10.24 μg/mL. The microbroth dilution method was used to determine the MIC of the two TCM monomers against *E. coli.* This method is recommended by the American Society for Clinical and Laboratory Standards (CLSI) for drug sensitivity tests.

### Effect of TCM Monomers on Growth and Biofilm Generation

Strains with higher levels of resistance were selected for the detection of biofilm formation. For this purpose, 96-well plates seeded with the monomers at 1/2 MIC, 1/4 MIC, and 1/8 MIC concentrations and a negative control containing only LB broth medium were used. After adjusting to a bacterial concentration of 0.5 McMurray, the strain was inoculated into the wells at a concentration of 1% inoculum per well. The plates were incubated for 48 h at 37°C, and the medium was replaced at 24 h. After incubation, the plates were washed with PBS and fixed with methanol for 15 min; the excess methanol was then discarded, and the plates were allowed to dry. The plates were then stained with 1% crystal violet for 5 min, washed to remove the unbound dye, and then dried; the crystal violet dye was dissolved in 33% (v/v) glacial acetic acid. The OD_630_ value was determined using a microplate reader. The readings were taken in triplicate per well and then averaged.

### Laser Scanning Confocal Microscopy Observation

For this experiment, the strain with the strongest biofilm formation ability was selected. On a six-well cell culture plate, a piece of cover slip treated with concentrated sulfuric acid was placed overnight, and the culture solution with different concentrations of TCM monomers was added to each well. The blank group contained only LB, and after adjusting to a bacterial concentration of 0.5 McMurray, each well was inoculated with 1% inoculum. The six-well plate was placed in a constant temperature incubator and incubated at 37°C for 24 h. After incubation, the medium was discarded. The biofilm-attached coverslips were rinsed with PBS (pH = 6.8) and fixed with 2.5% glutaraldehyde for 1.5 h; the coverslips were then rinsed with PBS to remove the fixative and stained with FITC-ConA (Sigma, United States) in the dark at 4°C for 30 min; after staining, the coverslips were washed with PBS. The coverslips were then stained with PI (Sigma, United States) in the dark at 4°C for 15 min. After staining, the coverslips were washed with PBS, sealed with an anti-fluorescence quenching agent, and placed under LSCM (Leica, Germany) for observation.

### Effect of TCM Monomers on the Expression of Genes Related to Biofilm Generation and the QS

The TRIzol (Invitrogen) method was used to extract total RNA from the *E. coli* culture 48 h after incubation with the TCM monomers. In accordance with the instructions given in the reverse transcription kit, a cDNA template was synthesized, and gDNA on total RNA was eliminated before cDNA synthesis. RT-PCR was performed according to the instructions of the Universal SYBR qPCR Master Mix Kit (Novizan Biotechnology Co., Ltd., Nanjing, China). Eight primers from Tsingke Biotechnology were used (Table 1), with *gapA* as an internal reference gene. The qRT-PCR reaction conditions were as follows: denaturation at 95°C for 2 min, 39 cycles of denaturation at 95°C for 15 s, annealing at 62°C for 15 s, and a final extension at 72°C for 20 s. After the reaction was completed, the sample was cooled to 65°C for 2 min, and the temperature was then slowly increased to 95°C at the rate of 0.2°C/s while continuously measuring the fluorescence.

**TABLE 1:** Details of RT-PCR primer sequences.

**Gene**	**Length (bp)**	**Primers**	**Sequence**	**References**
*hflX*	128	*hflX-F*	5′-TGTAGGTGAAGGTAAAGCAG-3′	This study
		*hflX-R*	5′-CACGACACTCGCACAAACGC-3′	
*fliA*	112	*fliA-F*	5′-GCTGGCTGTTATTGGTGTCG-3′	Chen et al., 2018
		*fliA-R*	5′-CAACTGGAGCAGGAACTTGG-3′	
*motA*	120	*motA-F*	5′-CTTCCTCGGTTGTCGTCTGT-3′	Chen et al., 2018
		*motA-R*	5′-CTATCGCCGTTGAGTTTGGT-3′	
*ompA*	152	*ompA-F*	5′-TCCAGAGCAGCCTGACCTTC-3′	This study
		*ompA-R*	5′-GCTGAGCCTGGGTGTTTCCT-3′	
*gapA*	104	*gapA-F*	5′-GAAATGGGACGAAGTTGGTG-3′	Congrui et al., 2013
		*gapA-R*	5′-AACCACTTTCTTCGCACCAG-3′	
*luxS*	116	*luxS-F*	5′-TGCCACACTGGTAGACGTTC-3′	Chen et al., 2018
		*luxS-R*	5′-TGATTGGTACGCCAGATGAG-3′	
*pfS*	169	*pfs-F*	5′-CGGCAACAGCCAGGAACTCA-3′	This study
		*pfs-R*	5′-GCGAAAATCCGCCACAACTT-3′	
*sdiA*	105	*sdiA-F*	5′-AGTCTGATGGCTCTGATGCG-3′	This study
		*sidA-R*	5′-CTTACCTTCCGCCGTCCATT-3′	
*ftsE*	81	*ftsE-F*	5′-AAAGTACCCTCCTGAAGCTGATCTGTG-3′	This study
		*ftsE-R*	5′-GCGTGATGTCATGGCCGCTAAAC-3′	
*ftsQ*	95	*ftsQ-F*	5′-GTTTCTTCTCGCCGCAATAAT-3′	This study
		*ftsQ-R*	5′-AACACGACCCAGCCGCTCACC-3′	

*The primers for the target genes were designed using the NCBI primer designing tool.*

### Data Analysis

The results of the drug sensitivity test were determined according to the CLSI standards (Clinical and Laboratory Standards Institute [CLSI], 2015). Relative expression levels of the target genes were calculated using 2^–ΔΔCq^ (where ΔCq = Cq_(target gene)_ − Cq_(reference gene)_, ΔΔCq = △Cq_(test)_ −△Cq_(calibrator)_) as described previously (Livak and Schmittgen, 2001). Statistical analysis was performed using SPSS software (version 19.0). The symbol ^∗^ indicates a significant difference at *p* < 0.05, while ^∗∗^ indicates a significant difference at *p* < 0.01.

## Results

### Antibiotic Resistance and TCM Monomer Sensitivity

As shown in Table 2, the six *E. coli* strains showed multiple degrees of multidrug resistance to seven antibiotics. Two strains showed resistance to TE, while three strains showed mild or intermediate resistance to CHL. CTX, GM, and CIP showed bacteriostatic effects against six strains among these strains, strain 2 showed a high level of multidrug resistance. A high level of drug resistance was also detected among several other strains tested. Strain 3 also showed a high level of drug resistance. Other strains showed similar levels of resistance, with a high level of resistance mainly to AMP and CFV. The MIC of berberine against the standard *E. coli* ATCC 25922 strain and the resistant strain were 1280 and 2560 μg/mL, respectively; the MIC of matrine against the standard *E. coli* ATCC 25922 strain and the resistant strain was 5120 μg/mL (Table 2).

**TABLE 2:** Antibiotic-resistance phenotypes and MIC (μg/mL) of *E. coli* strains.

**Strains**		**Medicine**								
		**AMP**	**CFZ**	**CTX**	**GM**	**TE**	**CIP**	**CHL**	**Berberine**	**Matrine**
Standard	Phenotype	S	S	S	S	S	S	S	–	–
	MIC	4	8	0.25	2	2	0.25	4	1280	5120
Strain 1	Phenotype	R	R	S	S	S	S	I	–	–
	MIC	128	256	0.5	4	4	0.25	16	2560	5120
Strain 2	Phenotype	R	R	S	S	R	S	R	–	–
	MIC	64	256	0.25	2	16	0.25	32	2560	5120
Strain 3	Phenotype	R	R	I	S	R	S	S	–	–
	MIC	64	256	2	2	128	0.25	8	2560	5120
Strain 4	Phenotype	R	R	S	S	S	S	S	–	–
	MIC	256	256	16	4	4	0.25	4	2560	5120
Strain 5	Phenotype	R	R	S	S	S	S	S	–	
	MIC	128	256	0.5	4	4	0.25	4	2560	5120
Strain 6	Phenotype	R	R	S	S	S	S	I	–	–
	MIC	64	128	0.5	2	4	0.25	16	2560	5120

*S, sensitivity; I, intermediate sensitivity; R, resistance.*

### Effect of TCM Monomers on the Growth of *E. coli*

**FIGURE 1:**
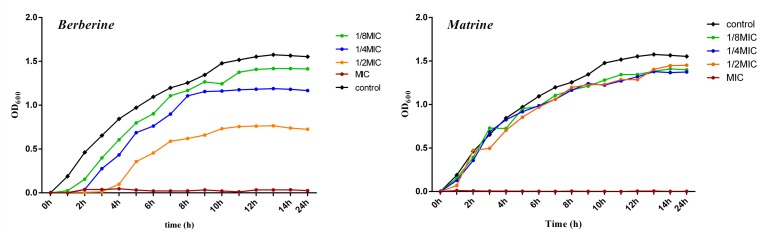
Growth curve of of *E. coli* treated with berberine and matrine at different concentrations.

As shown in Figure 1, the growth curve of *E. coli* in the blank group exhibited no lag period; the cells entered the logarithmic phase at around 1 h of incubation, entered the stationary phase at around 13 h of incubation, and then entered the death phase. Berberine showed a significant inhibitory effect on *E. coli*. *E. coli* growth was completely inhibited at a concentration of 1 MIC (1.28 mg/mL) berberine, while concentrations of 1/2 MIC (0.64 mg/mL), 1/4 MIC (0.32 mg/mL), and 1/8 MIC (0.16 mg/mL) delayed the growth of *E. coli* and its entry into the logarithmic phase. The cells entered the stationary phase at around 8 h, and the bacterial concentration then no longer increased at 13 h; the cells then began to die. The inhibition of *E. coli* growth was observed at all concentrations compared to the blank group without the drug. Matrine at different concentrations showed a weaker effect on the growth of *E. coli* than berberine, which mainly affects the stable growth period of *E. coli*. At 1 MIC (5.12 mg/mL), the growth of *E. coli* was completely inhibited; however, the other three concentrations did not significantly inhibit *E. coli* and showed similar effects.

### Effect of TCM Monomers on Biofilm Generation in *E. coli*

**FIGURE 2:**
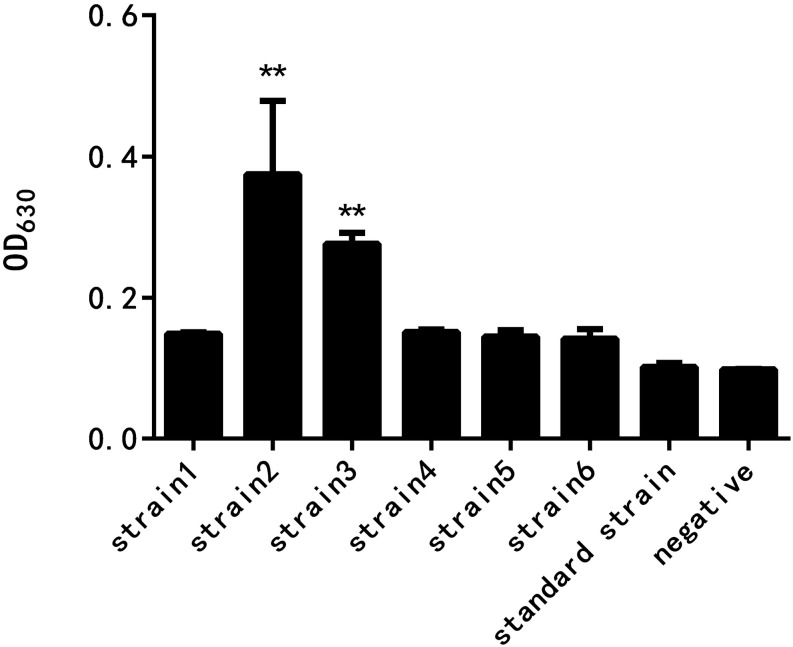
Comparison of the biofilm formation ability of *E. coli* at different drug resistance levels (^∗∗^ indicates a significant difference at *p* < 0.01).

As shown in Figure 2, the results of crystal violet staining showed that the biofilm formation abilities of the six resistant strains and the standard strain were different. The difference between the standard strain and the negative control group without the bacterial culture was not significant, indicating that the standard bacteria strain showed almost no biofilm formation. The drug-resistant strain 2 had the strongest biofilm formation ability, followed by the drug-resistant strain 3, and the difference was highly significant when compared with the negative control group. The biofilm formation of the other four drug-resistant strains was not significantly different from that of the negative control group.

**FIGURE 3:**
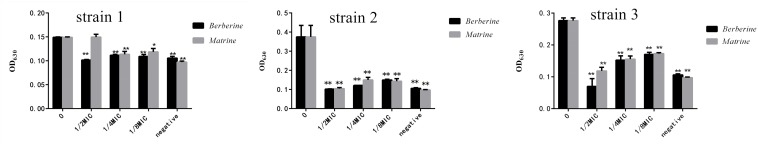
Effects of the two TCM monomers on biofilm formation of resistant *E. coli* (^∗^ indicates a significant difference at *p* < 0.05; ^∗∗^ indicates a significant difference at *p* < 0.01).

Strains 1, 2, and 3 with high drug resistance and biofilm formation abilities were selected to detect the thickness of the formed biofilm. As shown in Figure 3, berberine and matrine affected the biofilm formation of drug-resistant strain 1. However, the 1/2 MIC concentration of berberine showed more significant inhibition than matrine. The inhibitory effect of matrine at this concentration was not significantly different from that of the positive control group. The two monomers at other concentrations showed a similar biofilm inhibition effect; thus, 1/2 MIC is the optimal inhibitory concentration. Berberine showed the most obvious inhibitory effect on the biofilm formation of drug-resistant strain 3, followed by matrine.

### LSCM Observation

The coverslips with biofilm formed by the drug-resistant strain 2 after treatment with the TCM monomers were observed under LSCM at 40× magnification. FITC-ConA labels the bacterial biofilm polysaccharide matrix and emits green fluorescence at 488 nm wavelength, while PI labels the inner nucleus of the bacteria cells and emits red fluorescence at 535 nm excitation wavelength. The two fluorescence when superimposed give an orange yellow appearance to the stained parts. The results of LSCM showed that the blank control group formed a biofilm with strong adhesion and green fluorescence.

**FIGURE 4:**
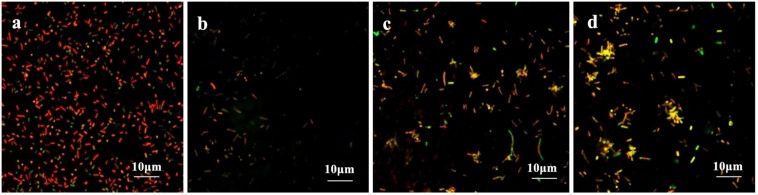
Comparison of the inhibitory effect of berberine on the biofilm formation of *E. coli*. **(a)** Untreated group, **(b)** 1/2 MIC concentration-treated group, **(c)** 1/4 MIC concentration-treated group, and **(d)** 1/8 MIC concentration-treated group.

**FIGURE 5:**
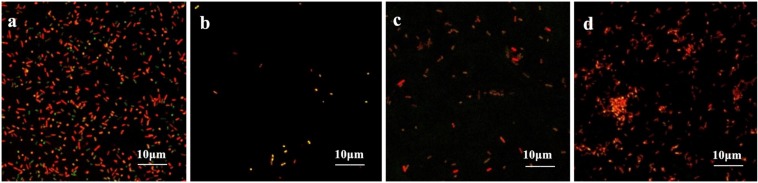
Comparison of the inhibitory effect of matrine on the biofilm formation of *E. coli*. **(a)** Untreated group, **(b)** 1/2 MIC concentration-treated group, **(c)** 1/4 MIC concentration-treated group, and **(d)** 1/8 MIC concentration-treated group.

Biofilm formation was significantly inhibited after berberine treatment, and the inhibition of biofilm formation by 1/2 MIC concentration was most obvious. The strains treated with berberine showed the following morphological changes: the cells were swollen and elongated, and showed a bright yellow fluorescence emitted from berberine itself (Figure 4). The treatment of *E. coli* with each concentration of matrine reduced biofilm formation. The 1/2 MIC concentration of matrine showed the most pronounced effect, followed by 1/4 MIC and 1/8 MIC concentrations (Figure 5).

### Effect of TCM Monomers on the QS System of *E. coli*

**FIGURE 6:**
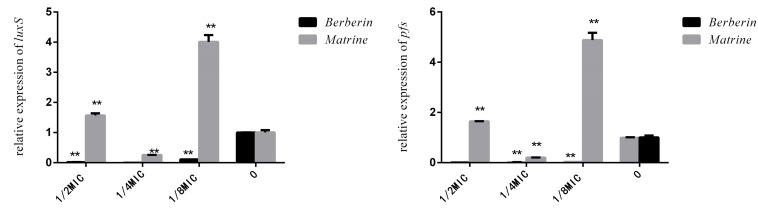
Effects of TCM monomers on the relative expression of the *luxS* and *pfS* genes in AI-2 synthesis (^∗∗^ indicates a significant difference at *p* < 0.01).

As shown in Figure 6, the downregulation of *luxS* and *pfS* genes by berberine treatment was more obvious than that by matrine treatment, and a highly significant difference was observed between all the treated groups and the untreated group. Matrine at 1/4 MIC downregulated the mRNA expression of these genes, with a highly significant difference, but the monomer at 1/2 and 1/8 MIC upregulated the expression of these genes. After berberine treatment at the sub-inhibitory concentration, the *pfS* gene of *E. coli* was hardly expressed. Matrine showed a significant downregulation of the relative expression level of the *pfS* gene at a concentration of 1/4 MIC, and the difference was highly significant when compared with the blank group (Figure 6).

**FIGURE 7:**
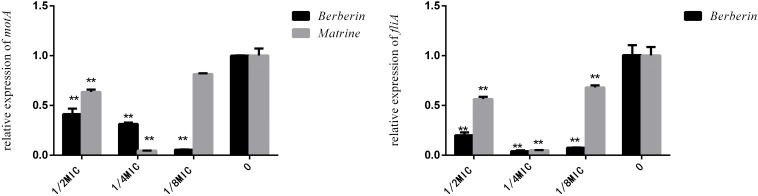
Effects of TCM monomers on the relative expression of the *fliA* and *motA* genes (^∗∗^ indicates a significant difference at *p* < 0.01).

Although berberine inhibited the expression of the *fliA* gene more strongly than matrine, the optimal concentration was 1/4 MIC. The two monomers at 1/4 MIC showed a large difference in the downregulation of *motA*, with matrine showing more pronounced downregulation of the gene than berberine (Figure 7).

**FIGURE 8:**
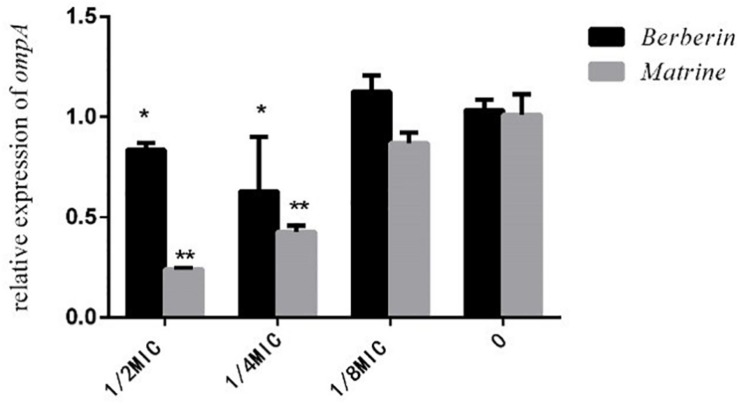
Effects of TCM monomers on the relative expression of the outer membrane protein *ompA* gene (^∗^ indicates a significant difference at *p* < 0.05; ^∗∗^ indicates a significant difference at *p* < 0.01).

As shown in Figure 8, both monomers downregulated the expression level of the outer membrane protein gene *ompA*, but matrine showed a better effect than berberine. The inhibitory effect of matrine at the 1/2 MIC concentration was most significant.

**FIGURE 9:**
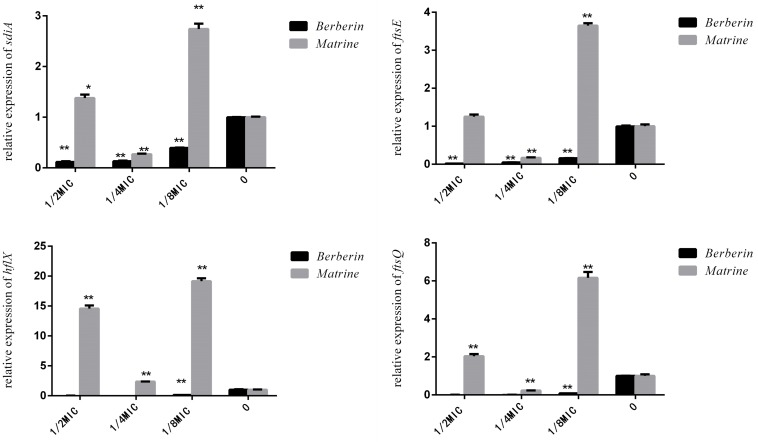
Effects of TCM monomers on the relative expression of the *sdiA*, *ftsQ*, *ftsE*, and *hflX* genes (^∗∗^ indicates a significant difference at *p* < 0.01).

As shown in Figure 9, matrine showed different effects on the expression of the *sdiA*, *ftsQ*, *ftsE*, and *hflX* genes in each concentration-treated group; only 1/4 MIC concentration showed downregulation of the expression of these genes, but the other concentrations of the monomer showed highly significant upregulation of the genes. After berberine treatment at different concentrations, the expression levels of these genes were downregulated, and the degree of downregulation was more obvious than that observed for matrine. Thus, the 1/2 MIC concentration of berberine was the optimal concentration for inhibiting gene expression, while 1/4 MIC was the optimal concentration for matrine.

## Discussion

### Effects of the Two TCM Monomers on the QS System of Drug-Resistant *E. coli*

**FIGURE 10:**
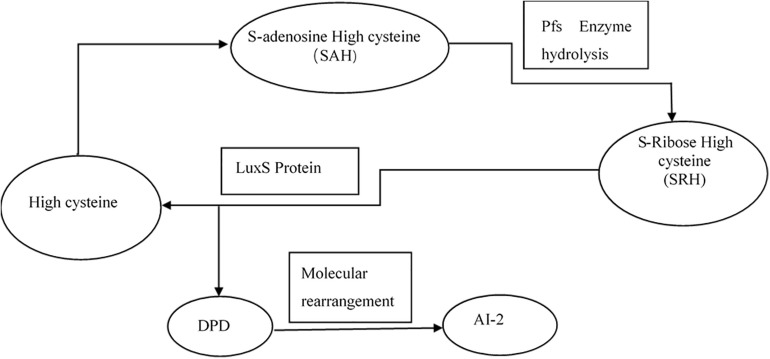
Synthesis of AI-2 (Vendeville et al., 2005).

**FIGURE 11:**
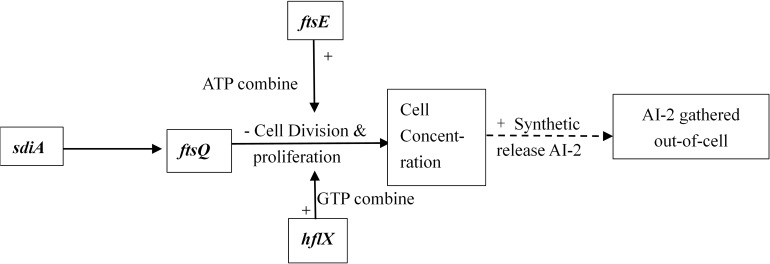
Adjustment pathway for the QS of *E. coli* (Vendeville et al., 2005).

**FIGURE 12:**
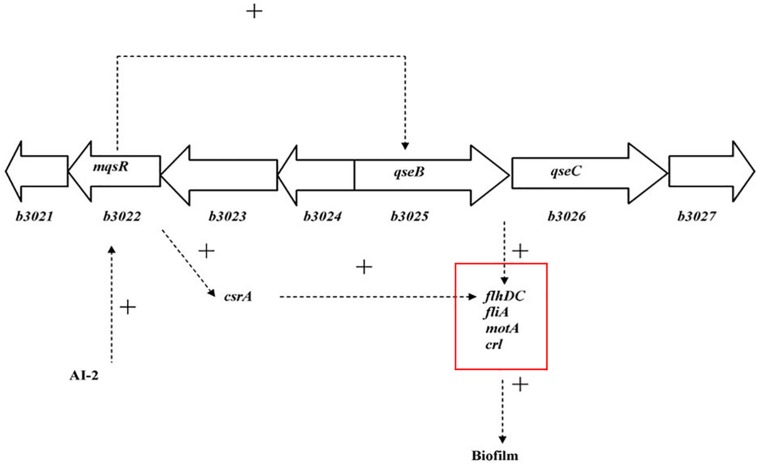
Model for the QS regulation of biofilms (Liu and Matsumura, 1994).

Interbacterial communication is accomplished by the secretion of a signaling substance called an autoinducer (AI) that binds to the receptor protein and induces bioluminescence (Kim et al., 2004). The QS system is classified into type I (LuxI/LuxR-mediated) and type II (LuxS/AI-mediated) systems, along with an oligopeptide-mediated QS system (mediated by AIP signaling molecules). The first two are commonly found in Gram-negative bacteria, while the latter is found in Gram-positive bacteria (Bassler et al., 1997). As shown in Figure 10, the *luxS* and *pfS* genes are regulatory genes for AI-2 formation in *E. coli*. In *E. coli*, when the QS inter-acoustic signal molecule AI-2 secreted by the bacteria reaches a certain concentration, it is transported into the cell by LuxS, and then phosphorylated; the signal molecule AI-2 binds to the receptor protein LuxS to regulate the formation and structure of the biofilm (Williams, 2002). Sun et al. (2014) found that the addition of LuxS or the addition of AI-2 signaling molecules to the culture medium increased the formation of biofilms by bifidobacteria. Motility is essential for the attachment and adhesion of *E. coli* biofilms, which control the adhesion, movement, and chemotaxis of *E. coli*. Bacterial chemotaxis is the key to bacterial colonization. *E. coli* lacks the *luxR* gene for the synthesis of AI-1, and its homologous gene *sdiA* regulates bacterial density and information exchange in *E. coli* while regulating the cell division-related genes *ftsQ*, *ftsE*, and *hflX* (Efimova et al., 2019). As shown in Figure 11, when the environment changes, the expression of *E. coli sdiA* gene is increased, which promotes the inhibition of the expression of the cell division inhibition gene *ftsQ*, with a subsequent decrease in the expression of *ftsE* and *hflX*, which weakens the ability of cells to bind ATP and GTP, respectively, and inhibits cell division (Vendeville et al., 2005). As shown in Figure 12, the QS signaling molecule AI-3 activates the two-component system *qseBC*, which is tightly linked to the *E. coli* flagellar-driven movement, in which the flagellar motor synthesis gene *fliA* and the motor-type gene *motA* regulate bacterial movement. The operon *motA* is controlled by *fliA*, which further affects the formation of biofilm (Liu and Matsumura, 1994).

The results of RT-PCR showed that berberine at the sub-inhibitory concentration significantly downregulated the expression of genes related to the QS system. All concentrations of berberine showed a good inhibition effect on biofilm formation; thus, berberine at all concentrations exhibited the best inhibition effect. In general, the relative expression of each gene in the 1/2 MIC concentration berberine treatment group was extremely low or almost zero. The 1/4 MIC concentration of berberine most obviously downregulated the flagellar synthesis gene *motA* and *fliA* and also the outer membrane protein regulatory gene *ompA*. We hypothesized that due to the extremely significant inhibition of the cell division genes *ftsE* and *ftsQ*, *E. coli* could not reach a certain density of growth in the medium. The effect of 1/2 MIC and 1/4 MIC concentration of berberine on the growth of *E. coli* may be related to this aspect. At the same time, the QS signaling molecule AI-2 synthesis genes l*uxS* and *pfS* showed almost no expression, which further inhibited the initiation of the QS. The LSCM results showed that after the strain was treated, the adhesion of the strain decreased and the morphology of the bacteria was changed. Specifically, the bacterial cells were swollen, thick, and elongated. Related studies have shown that berberine can change the permeability of the *E. coli* membrane, leading to a severe loss of calcium ions. Berberine shows a fluorescence reaction under ultraviolet light, which confirms that it can bind to DNA to enhance the fluorescence intensity of the dye (Hua, 2005). Studies have also shown that berberine exhibits its antibacterial activity through inhibition of the cell division protein *FtsZ* (Boberek et al., 2010). Berberine significantly downregulated the expression of QS-related genes at the low concentration of 1/8 MIC. Although the expression of *ompA* was upregulated, berberine could inhibit the formation of biofilm, and the concentration had little effect on the growth curve of *E. coli*. The discovery that berberine can inhibit QS initiation at lower concentrations with a minimal impact on growth is of great significance.

Different concentrations of matrine showed different degrees of inhibition on biofilm formation, but the results of RT-qPCR showed that the mechanism of action was different from that of berberine. Matrine at the 1/4 MIC concentration showed highly significant downregulation of nine genes. The 1/2 MIC concentration of matrine upregulated the cell division-related genes *sdiA*, *ftsQ*, *ftsE*, and *hflX*. However, the inhibition of the regulation of *ompA* by 1/2 MIC concentration was more obvious than that by 1/4 MIC concentration. Indicating that in the mechanism of inhibiting biofilm formation, 1/2 MIC concentration caused a weaker downregulation of other genes than that caused by 1/4 MIC and even upregulated these genes, but 1/2 MIC concentration of matrine still can significantly inhibit the formation of biofilm by *E. coli* with high drug resistance. The *ompA* gene acts as the main regulatory gene for the formation of *E. coli* outer membrane proteins, regulates the formation of *E. coli* biofilm and adhesion to host cells, and is closely related to the virulence of *E. coli.* Therefore, matrine inhibits the formation of biofilm of drug-resistant *E. coli.* The inhibition of *ompA* expression is predominant at the time of biofilm formation. Sharma et al. (2010) demonstrated that *sdiA* controls the downregulation of the *esp*, *eae*, and *flic* flagellation-related genes as well as the incremental expression of the *fts* operon *in vivo*. Therefore, although matrine upregulated the cell division-related genes, the synthesis of the signaling molecule AI-2 was inhibited, leading to the inhibition of the normal initiation of the QS.

### Effects of the Two TCM Monomers on the Formation of *E. coli* Biofilm

In the test of biofilm inhibition by berberine and matrine, the biofilm formation ability of the drug-resistant strain 2 was the strongest; this finding was consistent with the trend of drug resistance of the three strains. Thus, *E. coli* with high multidrug resistance and high drug resistance is more likely to form biofilms; this assumption is consistent with the conclusion reported by Chen et al. (2018) that the resistance rate and multidrug resistance rate of *E. coli* are directly proportional to its biofilm formation ability. In addition, the two TCM monomers showed more obvious inhibitory effects on strains with a strong biofilm formation ability. This is possibly due to the high concentration of QS signal molecules in such strains. Berberine mainly acts on the synthesis stage of AI-2 signaling molecules, thereby reducing the formation of secreted biofilms from the signaling molecules. The combined action of matrine on the outer membrane protein and the signaling molecule makes the bacteria less likely to aggregate and adhere. For the strain with a weak biofilm formation ability, the low drug concentration environment caused by the two monomers induced a stress pattern on bacterial biofilm formation; therefore, the two monomers are more targeted and have a better effect on strains with high levels of resistance. In a pre-laboratory study, the low concentration of the disinfectant benzalkonium bromide also showed an increase in the formation and growth of enterococci biofilm (Wu, 2018).

On the basis of the above results, it can be seen that berberine and matrine at sub-inhibitory concentrations showed good inhibition of biofilm formation by *E. coli* strains with high drug resistance, and a complete inhibitory effect was not observed. Although the effect of inhibition is not concentration dependent, the inhibition occurs at an optimum concentration. Berberine shows a stronger biofilm inhibition effect than matrine and causes a more significant downregulation of each gene. Both the monomers have different mechanisms of inhibition of biofilm formation at the level of gene transcription: berberine inhibits the formation of biofilms by inhibiting the QS system-related genes of drug-resistant *E. coli*, while matrine mainly inhibits the expression of the outer membrane protein gene *ompA*. It can be considered that the sub-inhibitory concentration of berberine and matrine can avoid the survival selection-driven pressure of *E. coli* and provide a basis for the weakening of *E. coli* resistance from the aspect of interbacterial population communication. The drug-resistant strains treated by the two monomers showed decreased adhesion, which is also an important factor for reducing the pathogenicity of *E. coli*.

## Data Availability Statement

The raw data supporting the conclusions of this manuscript will be made available by the authors, without undue reservation, to any qualified researcher.

## Author Contributions

TS, X-DL, JH, and CL conceived and designed the study. TS, X-DL, J-PZ, X-LZ, Y-JX, and Z-YO performed the experiments. TS, JH, CL, X-DL, J-LZ, and X-LZ analyzed the data and wrote the manuscript. All authors contributed to the editing of the manuscript.

## Conflict of Interest

The authors declare that the research was conducted in the absence of any commercial or financial relationships that could be construed as a potential conflict of interest.
